# Machine learning in the assessment and management of acute gastrointestinal bleeding

**DOI:** 10.1136/bmjmed-2023-000699

**Published:** 2024-02-19

**Authors:** Gaurav Bhaskar Nigam, Michael F Murphy, Simon P L Travis, Adrian J Stanley

**Affiliations:** 1 Translational Gastroenterology Unit, Oxford University Hospitals NHS Trust, Oxford, UK; 2 Transfusion Medicine, NHS Blood and Transplant, Oxford University Hospitals NHS Foundation Trust, Oxford, UK; 3 Kennedy Institute of Rheumatology, Nuffield Department of Orthopaedics, Rheumatology and Musculoskeletal Sciences and, Biomedical Research Centre, Oxford University, Oxford, UK; 4 Department of Gastroenterology, Glasgow Royal Infirmary, Glasgow, UK

**Keywords:** Gastroenterology, Blood transfusion, Endoscopy, Risk management

**Key messages**

- Machine learning can help to predict the risk of adverse outcomes and need for intervention in patients with acute gastrointestinal bleeding, allowing clinicians to intervene earlier and improve patient outcomes
- Use of machine learning in this context is still in its early stages, and further research is needed to refine and validate prediction models
- Interpretability and transparency of machine learning models are essential to gain the trust of clinicians and ensure that models are used appropriately in clinical practice
- Collaboration between clinicians and data scientists is critical to the successful implementation of machine learning in the management of acute gastrointestinal bleeding

Integration of machine learning has the potential to transform the management of acute gastrointestinal bleeding, but a transparent and collaborative approach will be key, argue Gaurav Nigam and colleagues

## Introduction

Acute gastrointestinal bleeding, which can affect both the upper and lower gastrointestinal tracts, poses a global healthcare challenge. Incidence rates for acute upper gastrointestinal bleeding have been reported to range from 15.0 to 172.0 per 100 000 person years, whereas rates for acute lower gastrointestinal bleeding have ranged from 20.5 to 87.0 per 100 000 person years.^1^ The UK has had a pioneering role in improving acute gastrointestinal bleeding patient care through national audits and novel risk score development for both acute upper and lower gastrointestinal bleeding.^2–4^ Results from a 2007 audit involving 6750 patients with acute upper gastrointestinal bleeding showed a high overall in-hospital mortality of 10%, with a striking 26% mortality among established inpatients who developed acute upper gastrointestinal bleeding.^2^ Similarly, a 2015 audit of 2528 patients with acute lower gastrointestinal bleeding reported a 3.4% overall in-hospital mortality and 18% mortality among established inpatients who developed acute lower gastrointestinal bleeding, often attributable to comorbidities rather than from severe haemorrhage.^3^

Recent years have seen efforts to enhance acute gastrointestinal bleeding management, encompassing changes in clinical practices, the development of new risk assessment scores, and improved medical and endoscopic treatments.^5^ Notably, interim results from a 2022 re-audit of acute upper gastrointestinal bleeding in the UK suggest a decline in overall in-hospital mortality to 8.2% (5.2% in new admissions and 19% in established inpatients) despite a more comorbid population compared with 2007.^6^

Patients with acute gastrointestinal bleeding often present with diverse comorbidities and bleeding causes, necessitating individualised management plans. Clinical uncertainties persist, including early discharge of patients at low risk of adverse outcomes, real-time prognostication for acutely ill individuals, and early identification of those needing specific interventions. Artifical intelligence (AI), particularly machine learning, holds promise in addressing these challenges.

AI is a form of autonomous learning for computers. The learning component, commonly referred to as machine learning, incorporates mathematical algorithms to build from unseen data and predict decisions for prespecified tasks. The data for machine learning based models need to be divided into three sets: training, validation, and test. The training and the validation sets help the model to recognise patterns and make associations while the test set is used for testing the model to minimise errors and improve accuracy. Some of the popular model choices include decision trees, gradient boosting model (XGBoost), k-nearest neighbors, regularised Cox regression, random survival forests, neural networks, and support vector machines.^7^ Table 1 provides a summary of various available machine learning models and their suitability for different tasks. Models based on machine learning have already been applied to subspecialist areas of gastroenterology and hepatology to aid diagnosis or prognostication.^8^ These models have also been shown to be useful for predicting and optimising blood transfusion.^9 10^ The models can use many more variables than conventional scoring systems and learn from serial data. This refines the algorithms with each new patient and thereby improves risk stratification to make more accurate predictions on new datasets.

In this narrative review, we provide insights into the current state of machine learning applications in acute gastrointestinal bleeding management, highlighting key themes, trends, and future directions.

**Table 1:** Descriptions of machine learning models and their suitability in prediction of acute gastrointestinal bleeding risk

Model	Description	Advantages	Suitability
Decision trees	Tree-like structure where internal nodes represent feature attributes, branches represent decisions, and leaves represent outcomes or labels	Easy to interpret and visualise, suitable for both classification and regression tasks	Appropriate when transparency and interpretability are essential, useful for initial insights into data
Gradient boosting model (XGBoost)	Ensemble learning technique that combines predictions of multiple weaker models (usually decision trees) to create a stronger predictive model	Excellent predictive performance, handles missing data effectively, provides feature importance rankings	High predictive accuracy in risk prediction tasks
k-nearest neighbors	Non-parametric, instance based learning method that classifies data points based on the majority class among their k-nearest neighbors	Simple to understand and implement, effective for small to medium sized datasets	Suitable for tasks where data points share similar characteristics, eg, patient risk assessment
Regularised Cox regression	Survival analysis method used for time-to-event prediction, considering both covariates and event times	Accounts for censoring, handles survival data, can incorporate covariate information	Particularly useful for predicting critical outcomes with time-to-event dependencies, eg, rebleeding
Random survival forests	Survival analysis technique using random forests to analyse survival data and estimate survival probabilities	Reduces overfitting risk compared with individual trees, handles high dimensional data	Beneficial for analysing survival data in patients with acute gastrointestinal bleeding and estimating time dependent risks
Neural networks	Deep learning models inspired by the human brain, composed of interconnected layers of artificial neurons	Captures complex, non-linear relationships, suitable for large datasets and diverse data types	Excels in tasks with intricate underlying patterns and substantial data requirements
Support vector Machines	Classification technique aiming to find an optimal hyperplane to separate data points into different classes	Effective for high dimensional data and adept at capturing complex associations	Valuable when dealing with complex, high dimensional data in risk prediction of acute gastrointestinal bleeding

## Current application of machine learning for acute gastrointestinal bleeding

### Risk prediction and outcome analysis

Various risk scores have been developed for patients with acute gastrointestinal bleeding; for example, Glasgow Blatchford score, full or pre-endoscopy Rockall score, AIMS65, Progetto Nazionale Emorragia Digestive score, Oakland score, and the more recently developed ABC score.^4 11^ These risk scores have been developed to assist clinical decision making and prediction of relevant clinical outcomes that include hospital based interventions (eg, need for transfusion, endoscopic treatment, interventional radiology, and surgery), rebleeding, and mortality. However, these risk scores exhibit variable performance, and a single score cannot predict all relevant outcomes.^12 13^

A systematic review of 14 observational studies that developed machine learning based models on acute gastrointestinal bleeding showed event prevalence of 2-20% for mortality, 11-21% for rebleeding, and 12-76% for need of intervention; each study included 147 to 2380 participants, with four to 50 variables (ie, demographic, laboratory, and clinical characteristics at presentation).^14^ These models performed well for predicting rebleeding, need for intervention, and mortality in patients with acute gastrointestinal bleeding. The same group then compared a model based on machine learning (XGBoost) with existing clinical risk scores (ie, admission Rockall, AIMS65, and Glasgow Blatchford score) for predicting a composite outcome of need for hospital based intervention or death within 30 days in patients with acute upper gastrointestinal bleeding.^15^ Their machine learning model, based on gradient boosting, performed better (area under the curve 0.90) than the three existing pre-endoscopic clinical risk scores (0.64-0.87) and could be used to identify patients who were at low risk and suitable for outpatient management more accurately (table 2).

**Table 2:** Performance of XGBoost machine learning model and clinical risk assessment scores on external validation study

Composite outcome*	External validation AUC (99% CI)	P value
XGBoost model	0.90 (0.87 to 0.93)	(Ref)
Glasgow-Blatchford score	0.87 (0.84 to 0.91)	0.004
Admission Rockall score	0.65 (0.60 to 0.71)	<0.001
AIMS65	0.64 (0.59 to 0.69)	<0.001

*Need for hospital based intervention or death within 30 days in patients with acute upper gastrointestinal bleeding.

AUC, area under curve; CI, confidence interval.

Another study analysed data from the electronic patient records of 5691 individuals who were admitted with acute gastrointestinal bleeding to an intensive care unit and developed an explainable machine learning based model that was better at predicting mortality (area under the curve 0.85) than the widely used APACHE IVa scoring system (0.80, P<0.001).^16^ The model also provided clinicians with the reasoning behind the outcomes to help them understand the results better. This machine learning based model is the first for acute gastrointestinal bleeding that used the SHAP (Shapley additive explanations) method to improve the trust of clinicians in such models. The SHAP method is one of several techniques to help explain how a machine learning based model works. Using this method, the model assigns a value, known as the Shapley value, to each input feature, indicating its importance in predicting the model output. This method uses the idea of coalition game theory, where variables form coalitions to create a more accurate prediction. The Shapley value is used to determine how fairly to distribute the prediction performance among the features. This approach helps to identify the most important variables in the model, improving accuracy and interpretability.^17^ This study was limited to intensive care.

### Predicting antithrombotic-associated acute gastrointestinal bleeding risk

The optimal timing of withholding and restarting antithrombotic medication in the context of acute gastrointestinal bleeding is a major challenge in clinical practice. An observational study on a cohort of patients who received various antithrombotic drugs showed modestly superior performance of two machine learning models, XGBoost and regularised Cox regression, in helping clinicians identify patients at risk for acute gastrointestinal bleeding.^18^ The study aimed to test the ability of three machine learning models (regularised Cox regression, random survival forests, and XGBoost) to look at time-to-event data for predicting acute gastrointestinal bleeding in patients with atrial fibrillation, ischaemic heart disease, or venous thromboembolism at six and 12 months after starting antithrombotic treatment. They also compared the performance of these models to the traditionally used HAS-BLED score (hypertension, abnormal kidney and liver function, stroke, bleeding, labile international normalised ratio, older age, and drug or alcohol use). The best performing model, regularised Cox regression, predicted acute gastrointestinal bleeding with an area under the curve of 0.67 at six months and 0.66 at 12 months. The XGBoost model resulted in identical area under the curves, and for the random survival forests model, the area under the curve values were 0.62 at six months and 0.60 at 12 months. The commonly used HAS-BLED model had an area under the curve of 0.60 at six months and 0.59 at 12 months. The performance of the machine learning models was modest (area under the curve was relatively low). All approaches, including the HAS-BLED score, were better at identifying patients at low risk.

### Predicting blood transfusion needs

In recent years, pivotal randomised controlled trials have shaped clinical practices, particularly by endorsing a more conservative approach to red blood cell transfusions in acute gastrointestinal bleeding, an area addressed in two significant randomised controlled trials.^19 20^ The focus has been on optimising transfusion policies with an evidence-based perspective, aligning with the principles of patient blood management. Initially successful in surgical settings, patient blood management has expanded into medical care to reduce blood product use and enhance patient outcomes.^21^ However, evidence supporting tailored transfusion strategies for acute gastrointestinal bleeding patients remains limited because existing risk scores do not precisely predict transfusion requirements.

Machine learning models have also been evaluated for their potential to predict the need for blood transfusions in patients with acute gastrointestinal bleeding. A large observational study used two publicly available intensive care unit datasets—MIMIC-III (Medical Information Mart for Intensive Care-III) and eICU-CRD (eICU Collaborative Research Database v 2.0)—to develop machine learning algorithms for predicting the need for blood transfusion in patients with acute gastrointestinal bleeding admitted to an intensive care unit.^10^ Given the intensive monitoring and detailed information available from intensive care unit datasets, initial observations with laboratory values, demographics, and clinical parameters over different time windows were used as covariates to develop these machine learning models. The best performing model resulted in an area under the curve of more than 0.80 using an observation period from the first five hours of intensive care unit admission and predicted the need for transfusion in the next 24 hours.^10^ Using a similar dataset (MIMIC-III), another group showed use of dynamic risk prediction by consolidating 62 demographic factors, laboratory tests, and clinical parameters into four hourly time intervals over the first 24 hours of admission.^9^ A long short-term memory model, a type of recurrent neural network, outperformed a regression based model (area under the curve: 0.65 *v* 0.56; P<0.001) in identifying patients with acute gastrointestinal bleeding who are at high risk of needing red blood cell transfusion in the intensive care unit. These types of models can help personalise the care of such patients using information in real time for dynamic risk prediction, which could be helpful to clinicians.

### Endoscopy image analysis and classification

Other than risk prediction, machine learning can be used for endoscopy image analysis. Endoscopy reporting uses classification systems, developed for various pathologies, to stratify patients based on their risk of needing an endoscopic intervention or of repeat bleeding, or both. One such classification system used in peptic ulcer bleeding is the Forrest classification.^22^ However, the endoscopist’s expertise affects their capacity for accurate classification, and experienced endoscopists have been suggested to be better at correct identification of at-risk features than trainee endoscopists.^23^

A single centre, retrospective cohort study explored the use of a deep learning model to classify still endoscopic images as per the Forrest classification for peptic ulcer bleeding.^24^ The authors used 2378 still images of peptic ulcer bleeding from 1694 patients to develop and a test machine learning model and compared its performance with expert and trainee endoscopists. The interobserver agreement of the machine learning model was moderate to substantial with expert endoscopists on the testing dataset (Cohen’s kappa coefficient: <0 indicating no agreement, 0-0.20 as slight, 0.21-0.40 as fair, 0.41-0.60 as moderate, 0.61-0.80 as substantial, and 0.81-1 as almost perfect agreement). The machine learning model’s accuracy was higher than that of a trainee endoscopist. These image analysis models based on machine learning have the potential to be used to aid clinical decision making for endoscopists.

### Integration of AI into electronic patient records for acute gastrointestinal bleeding

AI and machine learning could have a part in improving various aspects of clinical care for patients with acute gastrointestinal bleeding. Most of these studies have used retrospective data, either cross-sectional datasets or retrospective case linkage to electronic patient record. The widespread adoption of electronic patient records has opened up the possibility of integrating machine learning models into the electronic patient record platforms. However, the correct use of various risk stratification scores also relies on the ability to identify the patient subset for which these models need to be applied. Traditionally, clinical codes or manual chart reviews have been used to select the appropriate patient population, which is also known as phenotyping. AI and machine learning can assist in automating this process of phenotyping using clinical information presented in the electronic patient records.

Using health informatics, one study developed robust multiple natural language processing based approaches to identify patients with acute gastrointestinal bleeding in real time.^25^ Natural language processing is a form of machine learning technique that uses syntactic processing, semantic analysis, and the placement and sequencing of words in sentences and phrases, to extract data from narrative text. The authors evaluated the effectiveness of phenotyping algorithms based on electronic patient record data and compared it to the systematised nomenclature of medicine (SNOMED), a common system for classifying medical disorders in patients. The natural language processing based approach performed better than SNOMED (positive predictive value of 85% (95% confidence interval 83% to 87%) *v* 69% (66% to 72%); P<0.001) in identifying patients with acute gastrointestinal bleeding. This approach can aid the intuitive trigger of machine learning models embedded in an electronic patient record platform for risk stratification once the patient meets the phenotypic characteristics of acute gastrointestinal bleeding.

## Challenges and opportunities in advancing machine learning for acute gastrointestinal bleeding management

### Interpretability and explainability

While machine learning models may result in better performance, they typically sacrifice interpretability because of the underlying complexity. Clinicians prefer models that they understand and that correspond to their own experience and knowledge. This factor is key in the widespread use of score-card risk calculators based on statistical modelling in clinical practise, such as Glasgow Blatchford score and HAS-BLED, even though their quantitative performances may not be excellent. The domain of interpretability and explainability of machine learning is gaining significant research momentum with a focus on adding explainability features, such as those mentioned previously of Shapley values in published research.^17^

### Complementary role of machine learning models

These models should only be considered as a supportive tool and not as a replacement for clinical acumen and decision making. Given the modest performance of some of these machine learning models, they may be better suited for use as a supportive tool to enhance clinical decision making in the context of other clinical information, rather than relying solely on the model to make the decision. AI and machine learning has also got a potential to improve quality of clinical care, aid training of healthcare professionals, and be used in clinical trials.^24 26 27^

### Integration with clinical systems

To optimise the usefulness of machine learning methods in care for patients with acute gastrointestinal bleeding, seamless integration with electronic patient records and endoscopy reporting platforms is essential. Research has shown the potential of using different machine learning methods in specific clinical scenarios for patients with acute gastrointestinal bleeding. However, to enhance their use and clinical usefulness, aiming to improve patient outcomes, seamless integration of such AI and machine learning systems with electronic patient records and endoscopy reporting platforms is required. The results from various studies to date suggest that a single optimal machine learning system might not be possible; therefore, the creation of separate machine learning models may be required to predict specific outcomes of interest to clinicians and aid patient management. This could lead to the development of a clinical decision support tool, which collates well performing machine learning models for various outcomes of interest and embeds them on electronic patient record platforms. Nevertheless, one of the challenges lies in obtaining clinicians’ acceptance with AI and machine learning systems because their adoption and use require both a trust in the technology and a shift in traditional clinical practices. Furthermore, realising the full potential of machine learning in clinical practice may require substantial changes to hospitals' IT infrastructure, enabling real-time evaluation of machine learning predictions.

### Real-world application challenges

Considering the rapid evolution in technology supported by high quality evidence of the value of AI and machine learning in many aspects of medicine, including gastroenterology, its application in everyday clinical practice will soon become a reality. Its real-world application may present its own challenges including potential scepticism from clinicians and patient support groups as ethical and regulatory issues may arise. These systems will need to navigate the necessary complexities with data privacy, ownership, storage, and security. Notably, the performance of machine learning models is intrinsically tied to the quality of their training datasets, emphasising the importance of addressing disparities and minority classes in future prediction model development. Innovators, researchers, clinicians, and regulators will need to work together to find meaningful and responsible solutions to these challenges. Table 3 summarises important challenges, their description, and potential solutions in the use of machine learning in healthcare.

**Table 3:** Challenges, their description, potential solutions, and barriers for implementation in the use of machine learning (machine learning) in healthcare

Challenge	Description	Potential solution	Potential barriers for implementation
Data availability	Limited data for some patient populations, such as those with particular comorbidities, making training of models based on diverse populations challenging	Use rich, country-wide datasets and high quality datasets using electronic patient record through collaborative data sharing between large healthcare organisations	Barriers to data sharing, privacy concerns, and data ownership issues
Model validation	Difficulty in validating machine learning based models for risk assessment in real-world clinical settings requiring prospective data collection	Hybrid approaches that combine retrospective data analysis, simulation studies, external validation, and prospective data collection	Prospective data collection may be time consuming and costly, and challenging to obtain the necessary data
Clinical relevance	Translating machine learning based model results into clinically relevant and actionable information for healthcare professionals	Involving clinicians in the development of machine learning based models to ensure relevance and applicability to clinical practice	Ensuring alignment between clinical goals and model predictions, resistance to change in clinical practice, and communication barriers
Clinical workflow integration	Integrating machine learning based models into the clinical workflow without disrupting existing processes and adding value to decision making	Developing clinical decision support tools that incorporate the outputs of machine learning based models and provide actionable information in electronic patient record or on cloud platforms to improve efficiency and clinical workflow	Resistance to changes in clinical workflow, disruption of existing processes, and technical hurdles related to integration
Model complexity	Balancing model complexity to capture relevant information while maintaining interpretability and usability	Developing explainable models and selecting relevant features based on clinical inputs to reduce complexity	Difficulty in understanding highly complex models, resistance to model interpretation, and reluctance to use black-box models in clinical practice
Data privacy	Protecting patient privacy and ensuring the security of sensitive medical data when using machine learning based models in healthcare	Use of central data warehouses, anonymised datasets, encryption, and access control for file transfers and storage. Developing data sharing agreements between healthcare organisations and research institutions. Ensure compliance with data protection regulations, including data protection act, HIPAA and GDPR, to guarantee legal and ethical handling of sensitive medical data throughout collection, storage, and use	Compliance with data protection regulations, eg, HIPAA and GDPR, may entail significant implementation costs and require significant IT infrastructure updates and ongoing monitoring

GDPR, General Data Protection Regulation; HIPAA, Health Insurance Portability and Accountability Act.

## Conclusions and broader implications in clinical practice

Evidence supports the use of models based on machine learning in acute gastrointestinal bleeding for various purposes: optimising blood product use, facilitating early discharge of patients at low risk for adverse outcomes, providing real-time prognostication for patients who are acutely unwell, and aiding in the identification of patients requiring specific interventions. Machine learning models have the potential to address ongoing clinical uncertainties, such as accurately identifying patients at low and high-risk of adverse outcomes, determining optimal endoscopy timing, and understanding the impact of concurrent medication and comorbid conditions on management and outcomes.

**Figure 1:**
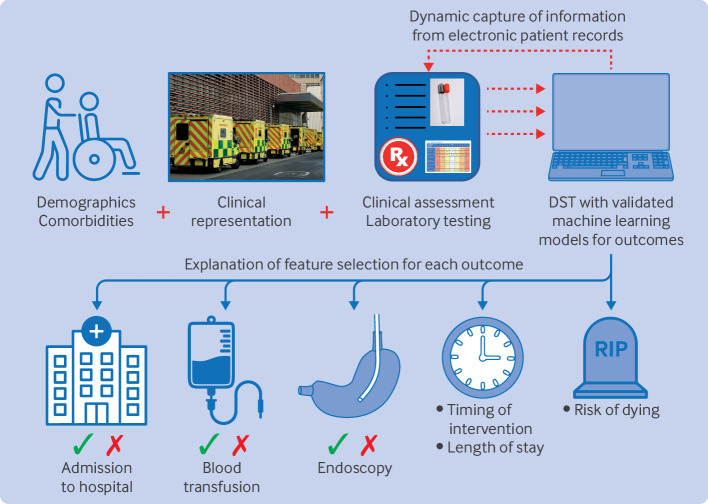
Proposed dynamic decision support tool (DST) for acute gastrointestinal bleeding using machine learning based risk prediction models

Machine learning can also contribute to the development of clinical decision tools, enabling individualised care plans for patients with acute gastrointestinal bleeding. By leveraging routinely collected clinical and electronic health record data, a dynamic decision support tool based on machine learning models can improve treatment outcomes through personalised care plans (figure 1).

Further research is warranted to investigate the use of national cross-sectional data, integrating endoscopic findings, interventions, and detailed analysis of electronic patient record datasets, to develop real-time clinical decision support tools. Subsequently, rigorous clinical trial assessments are necessary to evaluate the clinical impact of such interventions. The ultimate objective is to provide standardised, evidence based optimal management at the point of care. Successful implementation of these decision support tools could have broader implications beyond acute gastrointestinal bleeding, potentially benefiting other acute and chronic clinical conditions. However, the development and successful use of robust machine learning models in clinical practice necessitates access to larger and representative datasets. Additionally, addressing the associated challenges and involving all stakeholders in the development of such machine learning based models is crucial.

## Data Availability

No data are available.
